# Supported Palladium Nanoparticles Synthesized by Living Plants as a Catalyst for Suzuki-Miyaura Reactions

**DOI:** 10.1371/journal.pone.0087192

**Published:** 2014-01-29

**Authors:** Helen L. Parker, Elizabeth L. Rylott, Andrew J. Hunt, Jennifer R. Dodson, Andrew F. Taylor, Neil C. Bruce, James H. Clark

**Affiliations:** 1 Green Chemistry Centre of Excellence, Department of Chemistry, University of York, York, United Kingdom; 2 Centre for Novel Agricultural Products, Department of Biology, University of York, York, United Kingdom; Queen’s University Belfast, United Kingdom

## Abstract

The metal accumulating ability of plants has previously been used to capture metal contaminants from the environment; however, the full potential of this process is yet to be realized. Herein, the first use of living plants to recover palladium and produce catalytically active palladium nanoparticles is reported. This process eliminates the necessity for nanoparticle extraction from the plant and reduces the number of production steps compared to traditional catalyst palladium on carbon. These heterogeneous plant catalysts have demonstrated high catalytic activity in Suzuki coupling reactions between phenylboronic acid and a range of aryl halides containing iodo-, bromo- and chloro- moieties.

## Introduction

The use of plants to capture specific elements, particularly metals, from contaminated soils or aqueous waste has long been recognized as a potential method for environmental clean-up. [1], [2] There has only been a limited number of studies into platinum group metals (PGMs) accumulation by plants, with most work focusing on the capture of metals such as, nickel, zinc and cadmium. [3] Whilst efforts to use plants as a tool for remediation of contaminated areas has demonstrated some promise, the full potential of this technology has not yet been realized.

Metallic nanoparticles (MNP) are receiving considerable interest from the scientific community due to the remarkable properties they present compared to the bulk of the same metal. [4] Previous work in the area of sustainable MNP production using plant extracts, algae and bacteria to form bio-MNPs has shown promise. [5]–[8] For example, Lloyd *et al* has synthesised palladium catalysts using bacteria such as, *Geobacter sulfurreducens* and *Escherichia coli* that show catalytic activity in a variety of reactions including Heck couplings. [9], [10] Yang *et al* have also shown that viruses can be used to make bio-MNPs, where tobacco mosaic virus-templated palladium nanoparticles were used as effective catalysts for Suzuki reactions. [11] Unfortunately, there are certain limitations when using bacteria for MNP synthesis, for example, a reducing agent such as H_2_ is often required in order for nanoparticle formation. [11], [12] There is also the problem that bacteria may not be stable under certain reaction conditions, for instance, many bacteria will disintergrate at high (>60°C) temperatures. Sulphur is an essential constituent of microbial biomass and when cells disintegrate this sulphur will be released into the reaction consequently poisoning the catalyst [8].

It has been proposed that a possible route to synthesis of MNPs is through biological production by living plants. [13] Plants are known to uptake metal salts and are capable of reducing these metal ions to form crystalline nanostructures. [14] Such MNPs in plants have been described for Au, Ag and Cu. [15]–[17] After formation of MNPs, it is common that extraction from biomass is attempted by methods such as freeze-thawing, biomass incineration, and chemical leaching. However, some limitations of these activities are that they are laborious, energy intensive and can destroy the MNP structure thus interfering with the desired nano-material properties. [5], [18] This paper demonstrates the first production of palladium nanoparticles (PdNPs) by living plants and, as an advantage over extraction methods outlined above, their direct use as effective catalysts for a range of Suzuki-Miyaura coupling reactions. Pd is arguably the most ubiquitous metal currently used in organic synthesis. Whilst some investigations into the potential bioaccumulation of palladium by plants have been carried out, [19] to the best of the authors knowledge no studies have investigated the fate of the palladium once it is accumulated or any potential applications of these materials.

## Materials and Methods

### Materials

Palladium on carbon 10% (Pd/C), palladium acetate (Pd(OAc)_2_), potassium tetrachloropalladate (K_2_PdCl_4_) and all reagents and solvents used in this work were purchased from Sigma Aldrich and used as received.

### Growth of *Arabidopsis* Containing Palladium

Wild-type Arabidopsis ecotypes Col-0 were obtained from the Nottingham Arabidopsis Stock Centre at the University of Nottingham. Seeds were imbibed for 4 days at 277 K on agar plates containing 20 mM sucrose and half strength Murashige and Skoog salts. Seedlings were grown in 16 h of light with 125 mmol m^–2^ s^–1^ white light. For liquid culture experiments, individual seedlings were transferred to 100-mL conical flasks containing 20 mL of half-strength Murashige and Skoog medium and 20 mM sucrose. Plants were grown under 20 mmol m^–2^ s^–1^ light on a rotary shaker at 130 rpm. The medium was then replaced with 10 mM potassium tetrachloropalladate(II). The plants remained in the palladium solution for 24 hours. Samples were taken for TEM periodically to 24 hours, then remaining tissue was washed, dried and ground to a powder.

### Transmission Electron Microscopy

Plant leaf tissues were fixed in 2.5% (v/v) glutaraldehyde, 4% formaldehyde (v/v) in 50 mM phosphate buffer for 3.5 hours a secondary fix of 1% osmium tetroxide. Samples were then dehydrated through a 25–100% acetone series and infiltrated with Spurrs resin (Agar Scientific) (25, 50 and 75%) with overnight polymerisation at 343 K. Sections were mounted on 400 mesh thin-bar Athene grids (Agar Scientific), stained with saturated uranyl acetate and Reynolds lead citrate and viewed using a Tecnai 12 Bio Twin TEM operating at 120 kV.

### Measuring Nanoparticle Size

Nanoparticle cross-sectional diameter was measured from TEM images of the plant. A minimum of 100 (but often >250) nanoparticles were measured for each material.

### X-Ray Photoelectron Spectroscopy

XPS spectra were recorded on a Kratos Axis Ultra DLD photoelectron spectrometer using a hemispherical photoelectron analyser with a monochromatic AlKα X-ray source (75–150 W) and analyser pass energies of 160 eV (for survey scans) and 40 eV (for detailed scans). Samples were mounted using double-sided tape. Binding energies were referenced to the C 1 s binding energy 285 eV. Prior to analysis samples were degassed overnight at ultrahigh vacuum (<5×10^−10^ Torr). CasaXPS software was used to carry out analysis of the spectra.

### Formation of the Catalyst

Two catalysts were prepared from the plant material. To produce the catalysts the dried plant powder was pyrolised using the Netzsch STA 409 under N_2_ at 1 K min^−1^ to 573 K (300°C) (Pd-P-300) and 1073 K (800°C) (Pd-P-800) respectively.

### Testing Catalytic Activity

For the reaction of aryl halide with phenylboronic acid typical reaction conditions were used: [20] Into a 25 ml round bottom flask the following reagents were measured; aryl halide (0.6 mmol), phenylboronic acid (0.7 mmol), sodium bicarbonate (2 mmol) and tetrabutylammonium bromide (TBAB) (1 mmol). Ethanol (2 ml) and water (1 ml) was then added and the flask heated with stirring to 328 K. Once the flask had heated to the required temperature Pd catalyst was added, equivalent amount of catalyst was added to achieve a 0.12 mol % Pd concentration (based on aryl halide concentration). For control experiments no catalyst was added, also a reaction was run with plant material not containing palladium, 8 mg of material was used. The reaction was allowed to proceed for 20 hours at 328 K. Reaction was monitored by GC-FID using diethyl succinate as a standard. After 20 hours the reaction was allowed to cool. The cooled reaction mixture was filtered to remove the catalyst, undissolved sodium bicarbonate and TBAB. The filtered solution was purified by flash column chromatography using pentane as the solvent. Purified, isolated products were characterised by ^1^HNMR and ^13^CNMR obtained using a JOEL JNM-ECS400 NMR operating at 400 MHz and 100 MHz respectively, 1024 scans were taken for each sample.

## Results and Discussion

**Figure 1:**
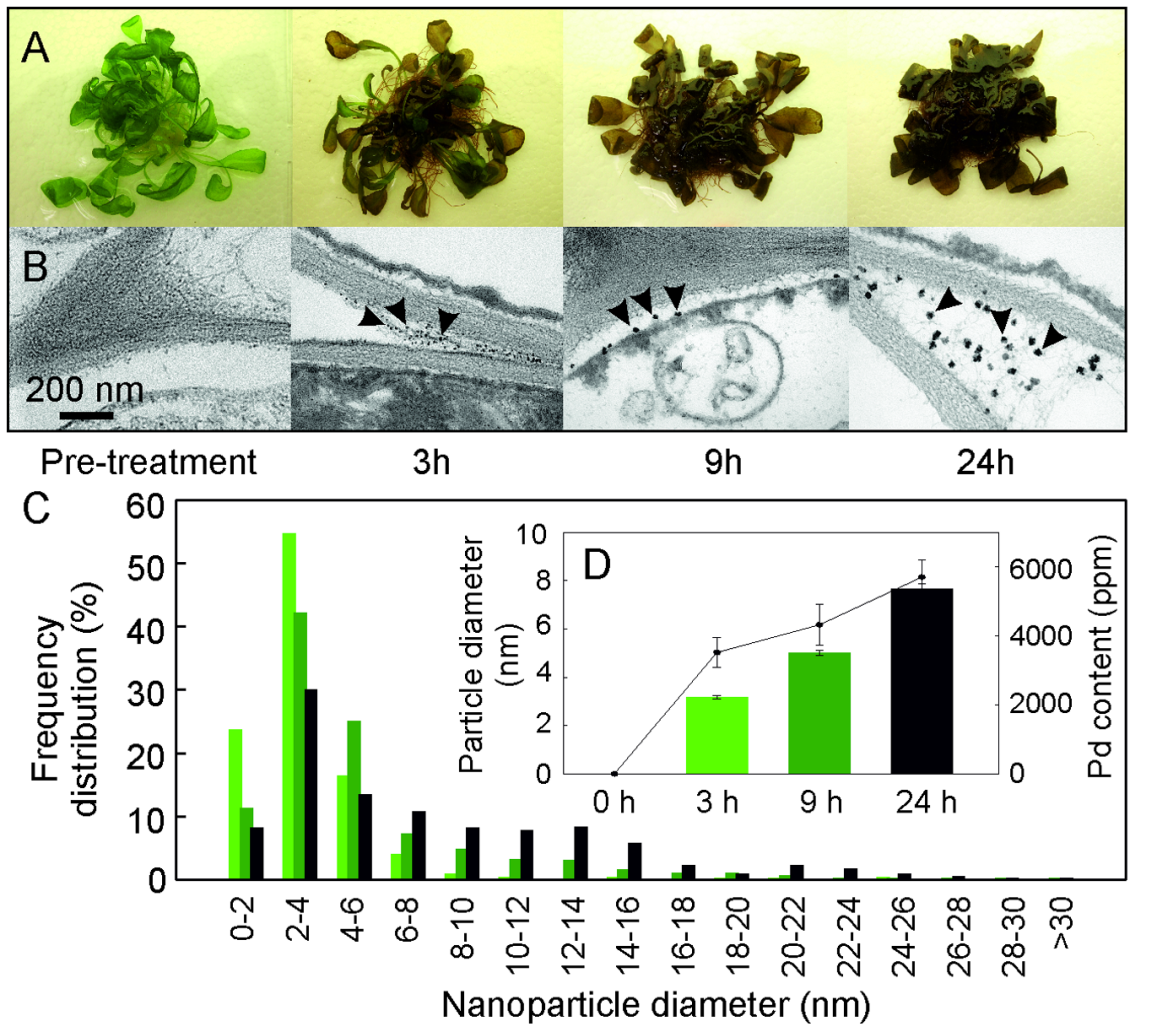
Pd uptake and PdNP formation in Arabidopsis: (A) appearance of 3-week-old plants 24 h after treatment with K_2_PdCl_4_. (B) TEM showing accumulation over time of PdNPs in cell wall corners. (C) Distribution of NP sizes in leaf tissue with time. (D) Mean NP diameter and Pd concentration with time.

The preparation and characterisation of Pd plant materials utilized techniques adapted from previously published work by Sharma *et al.* for the synthesis of gold nanoparticles. [21] For the work presented here, three-week-old, liquid culture-grown *Arabidopsis thaliana* (L.) (Arabidopsis) ecotype Col0 were dosed with an aqueous solution of K_2_PdCl_4_ over a period of 24 hours. Liquid culture was chosen over a soil-based system in order to easily control accurate dosing of metals. This method also allowed the plant material to readily be washed clean from the growth substrate after dosing, thus reducing contamination with non-plant material. After 24 hours from the time of dosing with palladium the plants appeared brown and wilted (Figure 1(a)), and were removed from the growth medium, washed, dried and ground to a powder. Transmission electron micrograph (TEM) images of sections of the plants revealed that within three hours of dosing, the plants had successfully formed well-dispersed, spherical MNPs with a mean profile diameter of 3 nm (Figure 1 (b)–(d)). Increasing concentrations and diameters of NPs were observed over time, with larger particles up to 32 nm formed by the end of the incubation (Figure 1 (c)–(d)). MNPs were distributed evenly adaxially to abaxially across the leaf (Figure S1), predominantly concentrated in the wall and apoplast regions of cell junctions. This distribution of MNPs across the leaf has not been reported before under the conditions used here. When the plants were grown in liquid culture, they were partially submerged, and the levels of cuticular wax on leaf surfaces appeared to be compromised. Together, these factors may have contributed to an increase in the passive uptake of metal ions through the leaf membranes. Inductively coupled plasma (ICP) analysis of palladium concentration in the plants over time showed the plants reached maximum palladium concentration after 18 hours of exposure (Figure 1 (d)).

**Figure 2:**
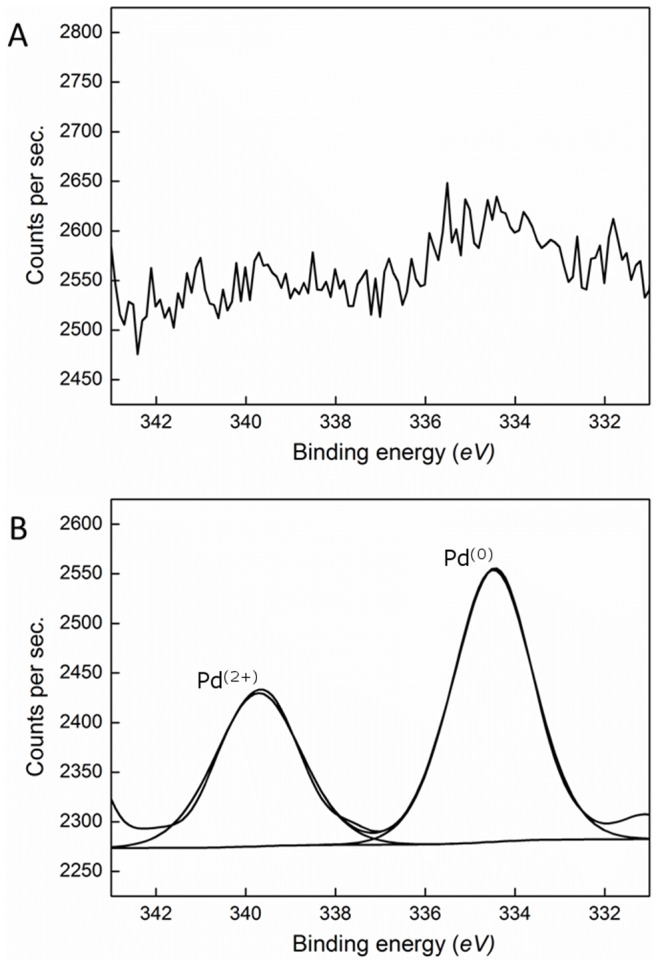
XPS spectra showing: a) undosed control plant, b) plant dosed with palladium for 24 h.

Analysis using X-ray Photoelectron Spectroscopy (XPS) on the dried Pd-plant material confirmed that the observed MNPs were palladium deposits. The spectra showed two chemically distinct spin-orbit pairs in the Pd 3d levels, centred at (I) 334.5 eV and (II) 339.7 eV binding energies, these peaks were not present in the control plant material (Figure 2). Peak (I) corresponds to fully reduced Pd^(0)^ nanoparticles, whilst peak (II) is due to unreduced Pd^(2+)^ ions. [22] The mechanism for metal reduction inside living plants is not yet understood but binding to carboxyl, amino and sulfhydryl groups are likely to be important in binding prior to reduction. A number of bacteria can reduce metals using enzymes such as the arsenate reductases and mercury reductases. [23], [24] However, similar enzymes have so far not been found in any plant species and the reduction of Pd^(2+)^ to Pd^(0)^ and formation of MNPs under the experimental conditions used here, could be a chemical, rather than biological, process.

**Figure 3:**
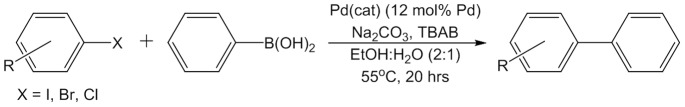
Suzuki-Miyaura C-C coupling reaction of arylhalide with phenylboronic acid.

Palladium catalyzed Suzuki-Miyaura C-C bond forming reactions are important methods for the synthesis of pharmaceutical intermediates and other high value molecules and were chosen to determine the catalytic activity of the plant catalysts (Figure 3). [25] In order to provide an effective support for the PdNPs the plant material was carbonized under nitrogen with a heating rate of 1 K min^−1^. This heating caused the diminution of heteroatoms such as oxygen and hydrogen as volatile gaseous emissions, resulting in the formation of a stable, carbon support that could withstand the reaction conditions. To determine the effect of carbonization temperature on PdNP state, and catalytic activity, the plant material was heated to 300°C or 800°C to produce materials named Pd-P-300 and Pd-P-800 respectively. During carbonization thermal gravimetric-infrared (TGIR) analysis of the material showed a 45% mass loss between 100–300°C, attributed to the loss of water and carbon dioxide (Figure S2). These catalytic materials contained a palladium concentration of 15%, determined by ICP, with the remaining 85% of the material made up predominantly of carbon and oxygen. The XPS analysis of Pd-P-300 showed the appearance of PdO after carbonisation of the material (Figure S3 and Table S1). The presence of PdO could be attributed to interaction between the Pd^(2+)^ and oxygen that is given off during heating. Table S1 also shows the XPS specta for palladium species on Pd/C, results showed that there are no Pd^(0)^ species present, only Pd^(2+)^ and PdO, this may result in a difference in reactivity of Pd/C and Pd-P-300.

**Figure 4:**
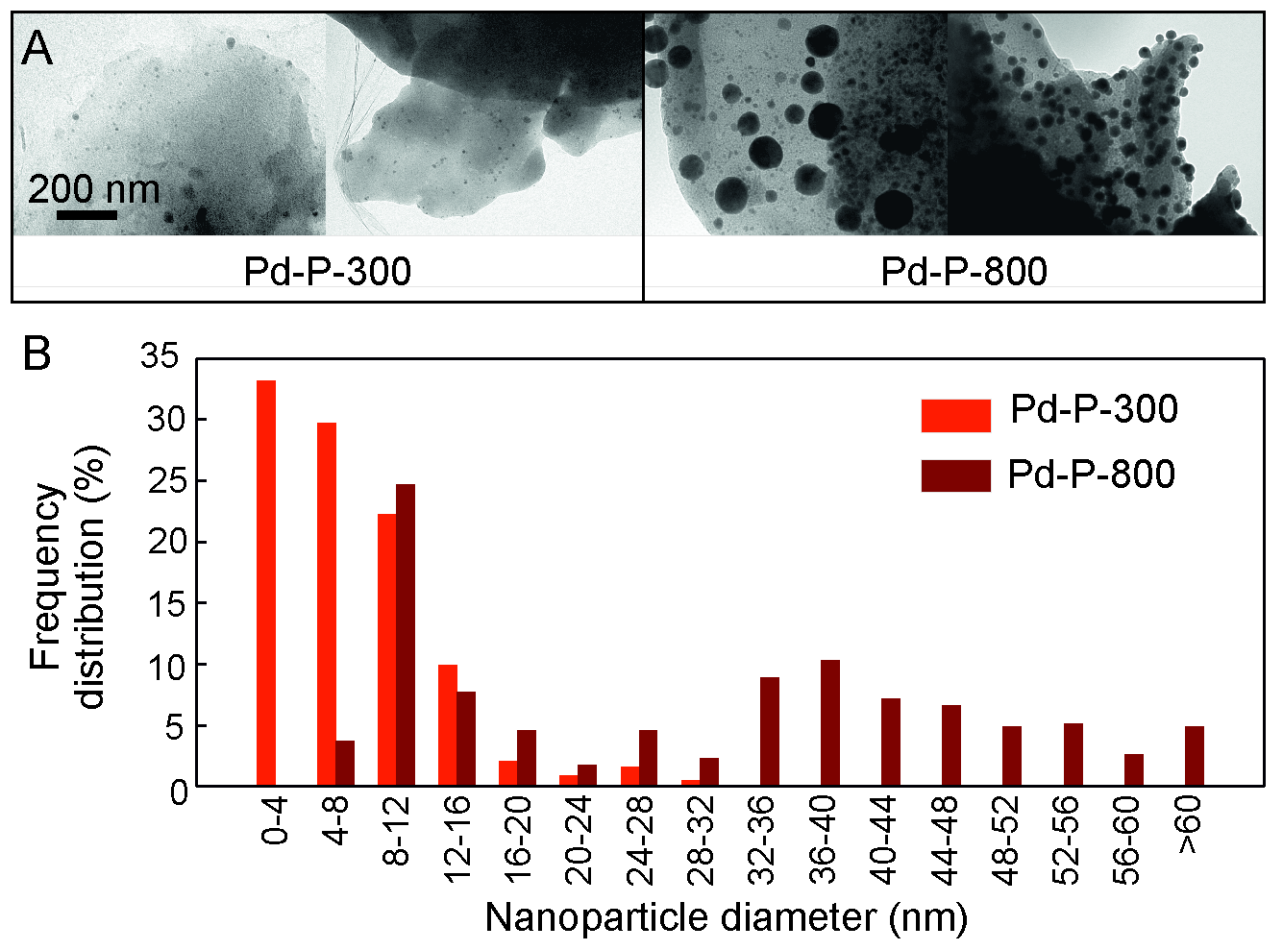
(A) TEM showing PdNPs in Pd-P-300 and Pd-P-800. (B) Distribution of PdNP sizes in the plant catalysts.

Analysis using TEM revealed highly dispersed, spherical palladium NPs in both catalytic materials (Figure 4). Further examination of Pd-P-300 revealed the mean cross-sectional diameter and frequency distribution of PdNPs was broadly similar to the pre-carbonized samples. However, for Pd-P-800, a marked shift in NP size was observed, with a 400% increase in mean diameter and wider frequency distribution. This indicates that between 300°C and 800°C PdNPs have diffused through the carbon support to form larger aggregations. This observation is consistent with previous observations by others, including Joo *et al*. and Jiang *et al.* [26], [27].

The driving force for NP aggregation comes from the higher chemical potential and increased surface free energy of metal atoms in small NPs in comparison with bulk metal surfaces. [28] This often leads to the deactivation of heterogeneous or supported NP catalysts and therefore may affect the activity of Pd-P-800. Initial reactions carried out using Pd-P-800 catalysts were unsuccessful resulting in no product likely due to the large PdNPs present and no further work was carried out with this material. Control reactions also exhibited no conversion without catalyst and when dried and carbonized plant material (containing no palladium) was used.

To test the activity of Pd-P-300 initially various temperatures, bases (organic or inorganic), solvents and mixtures of solvent were screened in order to determine the optimal reaction conditions (Table S2). The reaction of iodobenzene (1 mol eqv) with phenylboronic acid (1.2 mol eqv) in the presence of Na_2_CO_3_ (3 mol eqv) and tetrabuthylammonium bromide (TBAB) (2 mol eqv) in a solvent mixture of EtOH-H_2_O (2 ml:1 ml) at 55°C resulted in an isolated yield of >99% of the coupling product (Table 1, entry 1), and these reaction conditions were chosen for further experiments. TBAB was used in this reaction as a phase transfer agent and a stabiliser for the PdNPs, [29] previous work has shown that the presence of TBAB in Suzuki reactions facilitates the reaction [30].

**Table 1:** Suzuki-Miyaura coupling of aryl halides with phenylboronic acid under Pd-P-300 catalysis.[a]

Entry	R	X	Yield (%)[b]
1	H	I	>99 (91)[c] (>99)[d]
2	Cl	I	98
3	4-C(O)CH3	I	>99
4	3-NO2	I	93
5	4-C(O)CH3	Br	98
6	2-C(O)CH3	Br	94
7	4-NO2	Br	79
8	2-NO2	Br	>99
9	4-CN	Br	93 (60)[c] (78)[d]
10	3-CN	Br	>99
11	4-CN	Cl	81 (9)[c] (35)[d]

[a] *Reaction conditions:* aryl halide (0.6 mmol), phenylboronic acid (0.7 mmol), TBAB (1 mmol), Na_2_CO_3_ (2 mmol), Pd-P-300 (12 mol % Pd) in EtOH:H_2_O (2 ml:1 ml), NMR characterisation of products available in supporting information.

[b] Isolated yields after column chromatography with *n*-pentane.

[c] Isolated yield for Palladium on Carbon 10%.

[d] Isolated yield for palladium acetate.

Using this catalytic system Pd-P-300 was successfully applied to a range of other Suzuki-Miyaura reactions (Table 1). Yields were consistently high for the different aryliodides tested (Table 1, entries 1–4). The Pd-P-300 was also successful at catalyzing reactions involving a wide range of arylbromides (entries 5–11) and even a reaction with an arylchloride (entry 12) which are notoriously more difficult to react than the iodo-containing reagents. [31] This activity is excellent when compared against other non-conventional palladium catalysts. [11], [20], [32] As an example, Suzuki reactions carried out by Søbjerg *et al.*, under the same conditions, using bio-Pd^(0)^ formed using the gram-negative proteobacteria *Cupriavidus nectar* failed to show any reaction for the bromoarene tested (coupling using chloroarenes was not attempted). [20] In comparison to commercially available catalyst Pd on carbon 10% (Pd/C) and Pd(OAc)_2_, the reactivity of Pd-P-300 was superior in the case of all the reactions tested which is highly promising for the future of this material (Table 1, entry 1, 9 and 11).

## Conclusion

In summary, this work has demonstrated the ability of Arabidopsis to produce PdNPs in a relatively simple way without the requirement for toxic chemicals or energy intensive processes. These PdNPs show excellent catalytic activity across a range of Suzuki-Miyaura coupling reactions involving I, Br and Cl leaving groups, also producing higher yields than commercial Pd catalyst.

This method of plant-mediated MNP synthesis may represent a greener alternative to many other techniques. There is still further work to undertake, namely testing plant species more suited to in-field application, soil-based systems and the use of waste streams containing Pd to act as feedstocks.

## Supporting Information

Figure S1 Mean nanoparticle diameter in cells from adaxial to abaxial leaf surface. TEM sections from 3-week-old, liquid culture grown Arabidopsis plants were treated with 10 mM potassium tetrachloropalladate and the mean palladium nanoparticle profile areas measured across the leaf over time.
(TIF)

Figure S2 (A) Thermal gravimetric analysis of plant material pyrolisis to 300°C (B) DSC signal showing significant mass loss (C) Infra-red spectrum of emission at (B).
(TIF)

Figure S3 Pd XPS spectra of Pd-P-300.
(TIF)

Figure S4 Biphenyl ^1^H NMR and ^13^C NMR.
(TIF)

Figure S5 4-Chlorobiphenyl ^1^H NMR and ^13^C NMR.
(TIF)

Figure S6 4-Acetylbiphenyl ^1^H NMR and ^13^C NMR.
(TIF)

Figure S7 2-Acetylbiphenyl ^1^H NMR and ^13^C NMR.
(TIF)

Figure S8 4-Nitrobiphenyl ^1^H NMR and ^13^C NMR.
(TIF)

Figure S9 2-Nitrobiphenyl ^1^H NMR and ^13^C NMR.
(TIF)

Figure S10 4-Cyanobiphenyl ^1^H NMR and ^13^C NMR.
(TIF)

Figure S11 3-Cyanobiphenyl ^1^H NMR and ^13^C NMR.
(TIF)

Table S1 Composition palladium present in carbonised palladium plant (Pd-P-300) from XPS spectra.
(DOCX)

Table S2 Screening conditions to find optimal conditions for Suzuki reactions.
(DOCX)

Table S3 ^1^H NMR and ^13^C NMR data for isolated products.
(DOCX)
