# Supplementary figures and images for: Supported Palladium Nanoparticles Synthesized by Living Plants as a Catalyst for Suzuki-Miyaura Reactions
Source: PLoS One. 2014 Jan 29;9(1):e87192. doi: 10.1371/journal.pone.0087192 (PMC3906157; doi:10.1371/journal.pone.0087192)

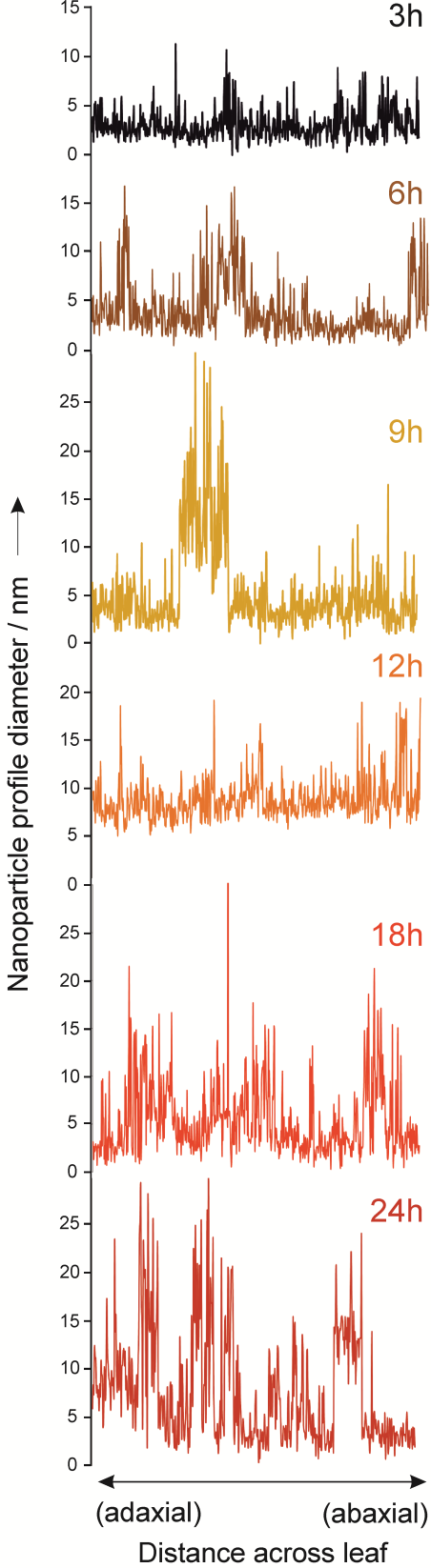

Supplement: Figure S1 — Mean nanoparticle diameter in cells from adaxial to abaxial leaf surface. TEM sections from 3-week-old, liquid culture grown Arabidopsis plants were treated with 10 mM potassium tetrachloropalladate and the mean palladium nanoparticle profile areas measured across the leaf over time. (TIF) [file pone.0087192.s001.tif]

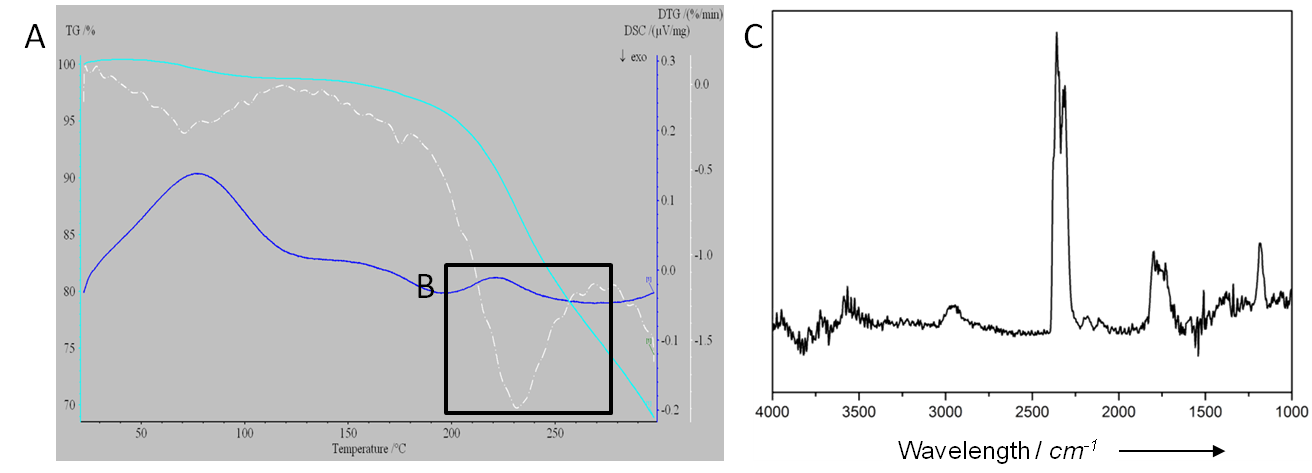

Supplement: Figure S2 — (A) Thermal gravimetric analysis of plant material pyrolisis to 300°C (B) DSC signal showing significant mass loss (C) Infra-red spectrum of emission at (B). (TIF) [file pone.0087192.s002.tif]

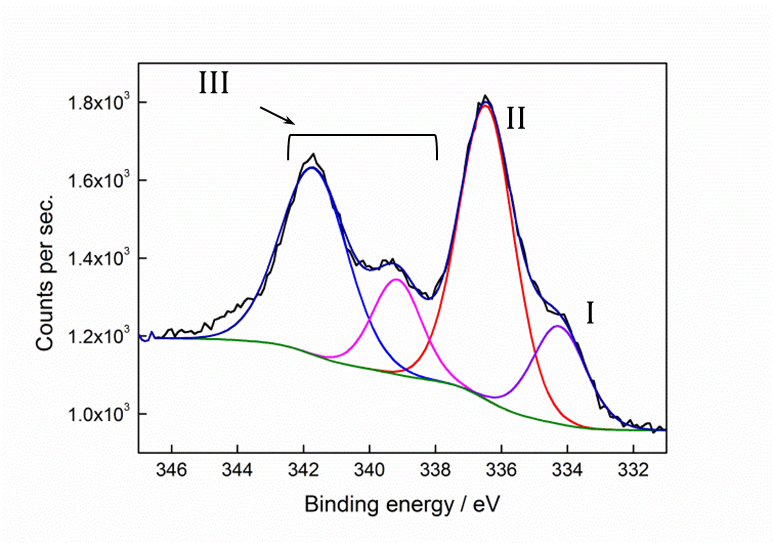

Supplement: Figure S3 — Pd XPS spectra of Pd-P-300. (TIF) [file pone.0087192.s003.tif]

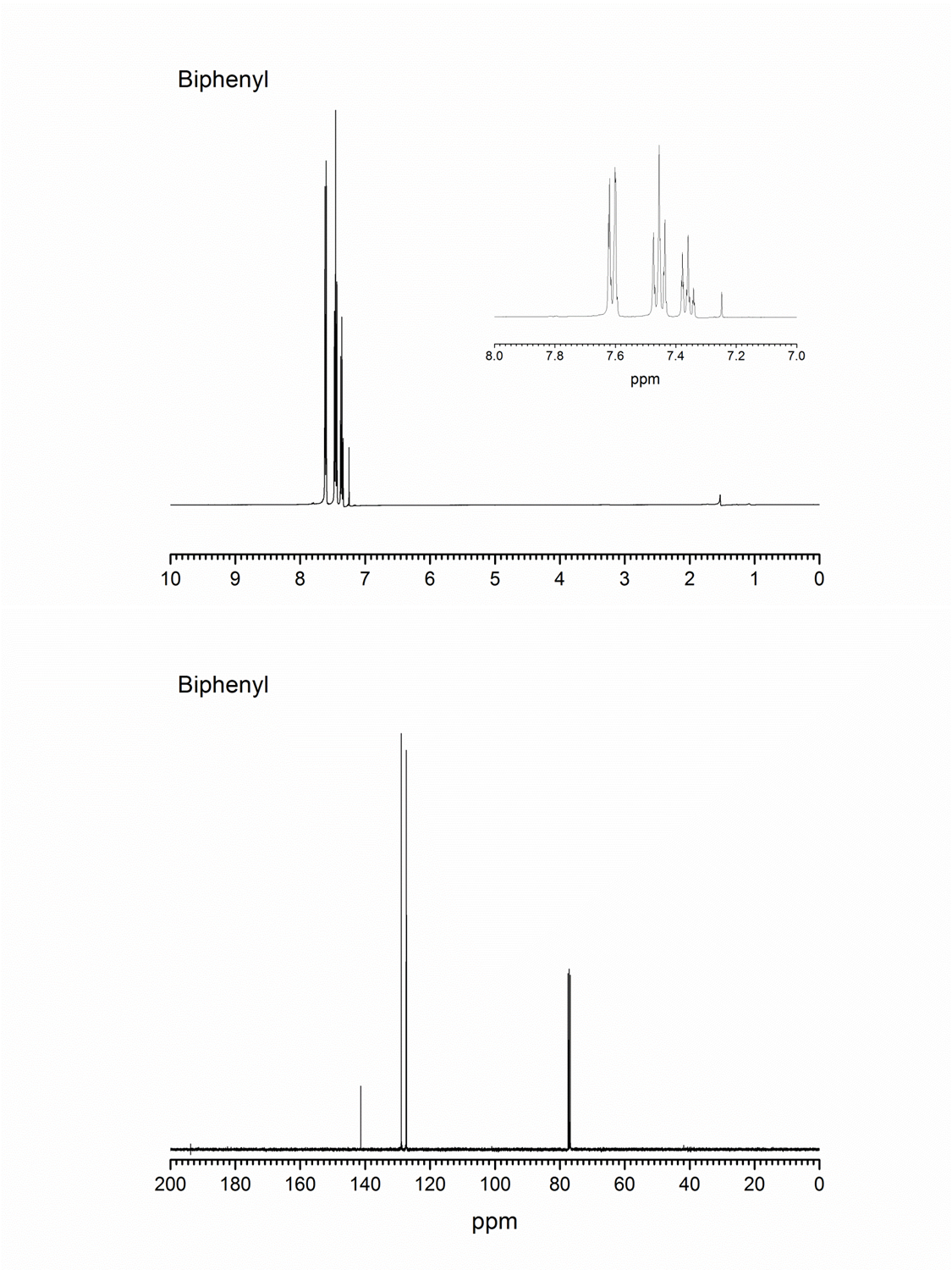

Supplement: Figure S4 — Biphenyl 1H NMR and 13C NMR. (TIF) [file pone.0087192.s004.tif]

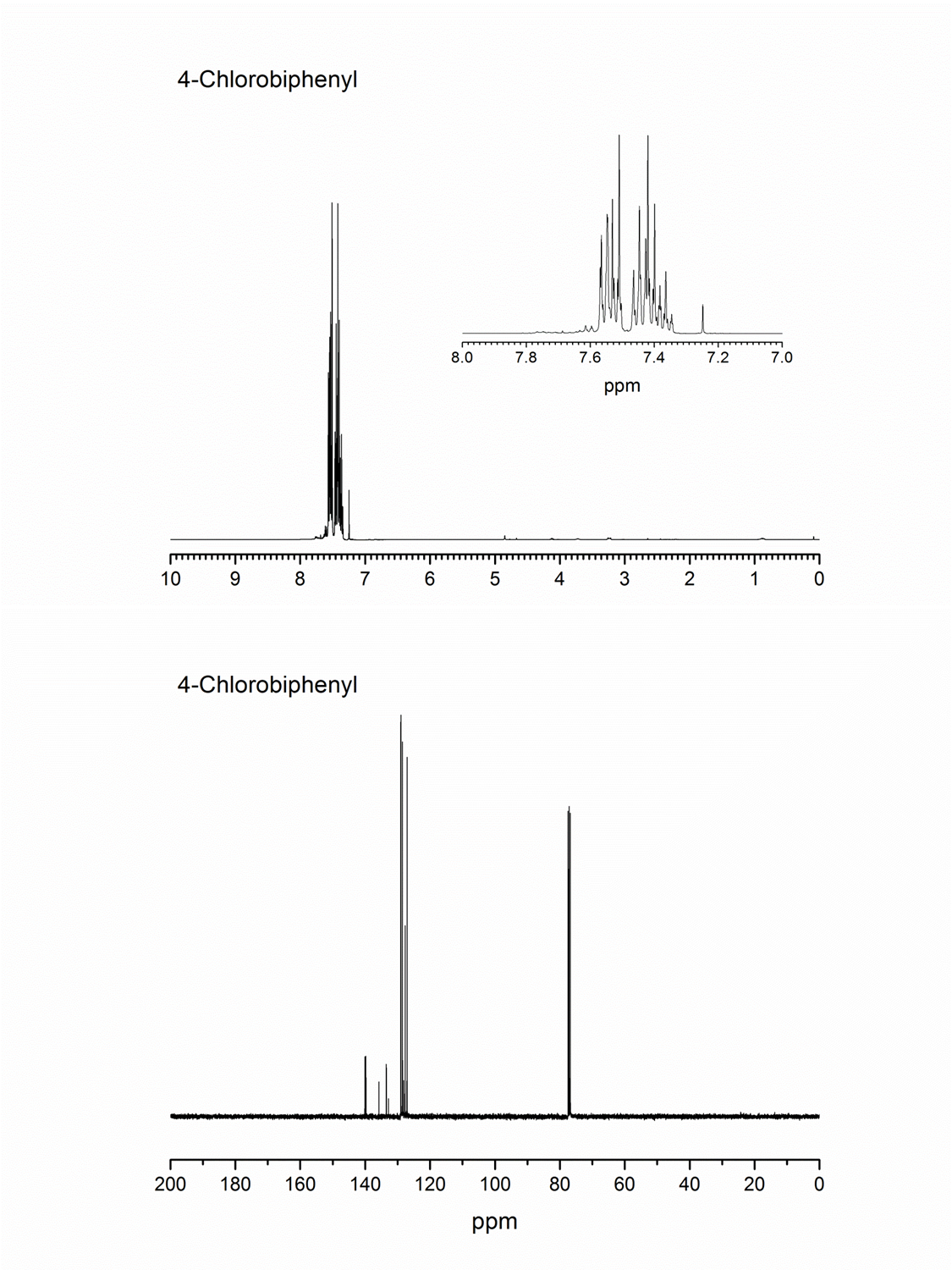

Supplement: Figure S5 — 4-Chlorobiphenyl 1H NMR and 13C NMR. (TIF) [file pone.0087192.s005.tif]

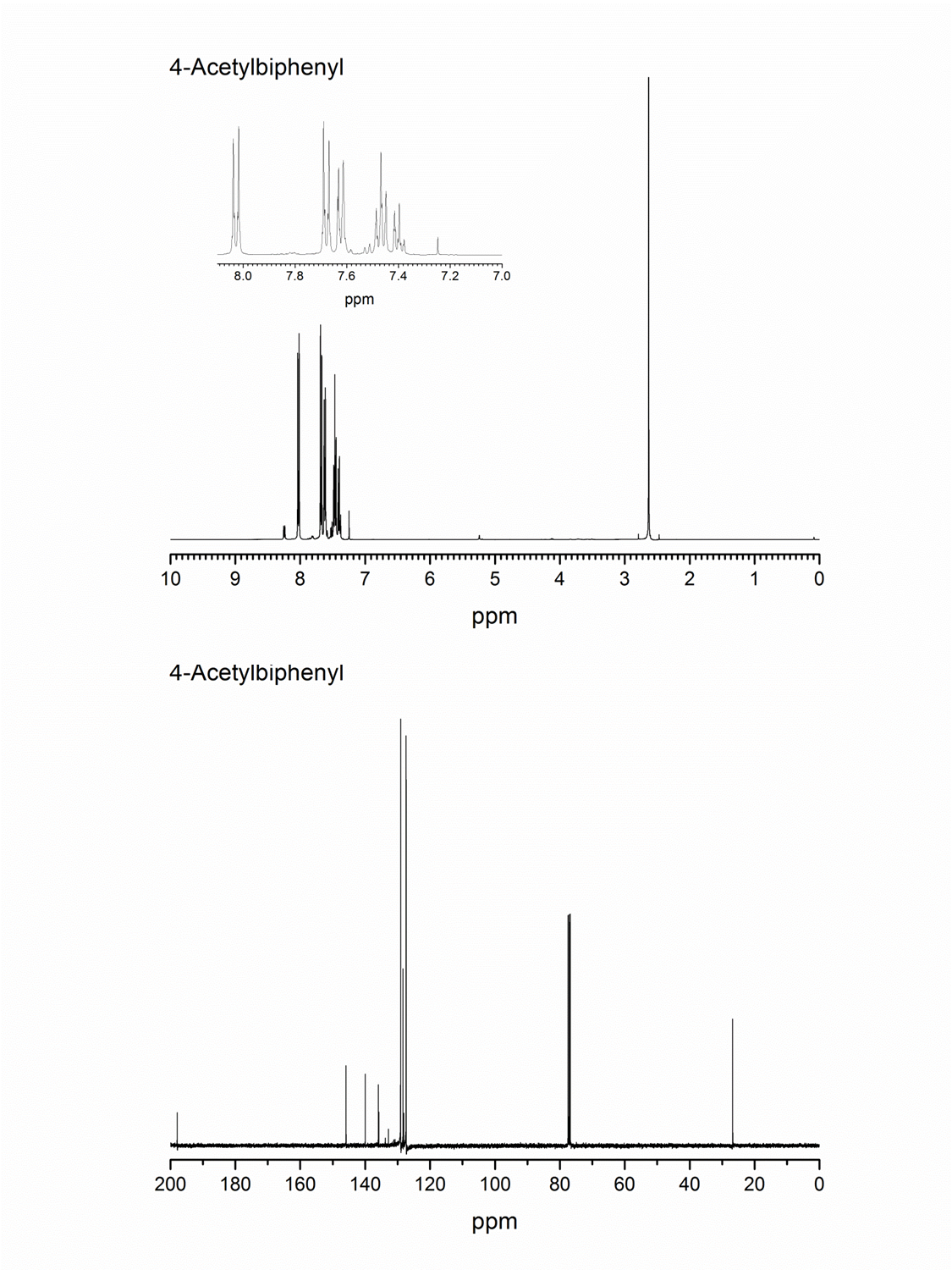

Supplement: Figure S6 — 4-Acetylbiphenyl 1H NMR and 13C NMR. (TIF) [file pone.0087192.s006.tif]

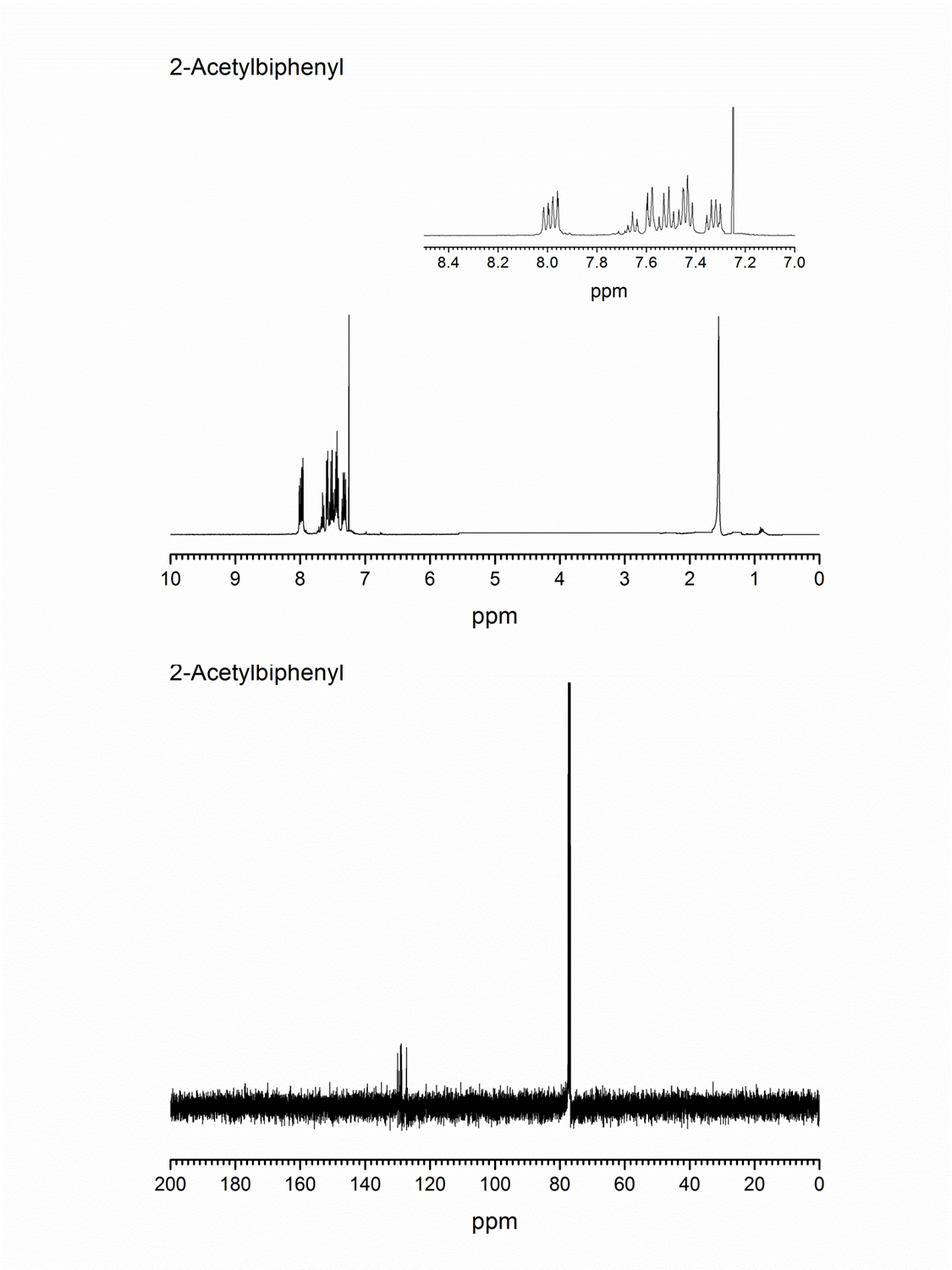

Supplement: Figure S7 — 2-Acetylbiphenyl 1H NMR and 13C NMR. (TIF) [file pone.0087192.s007.tif]

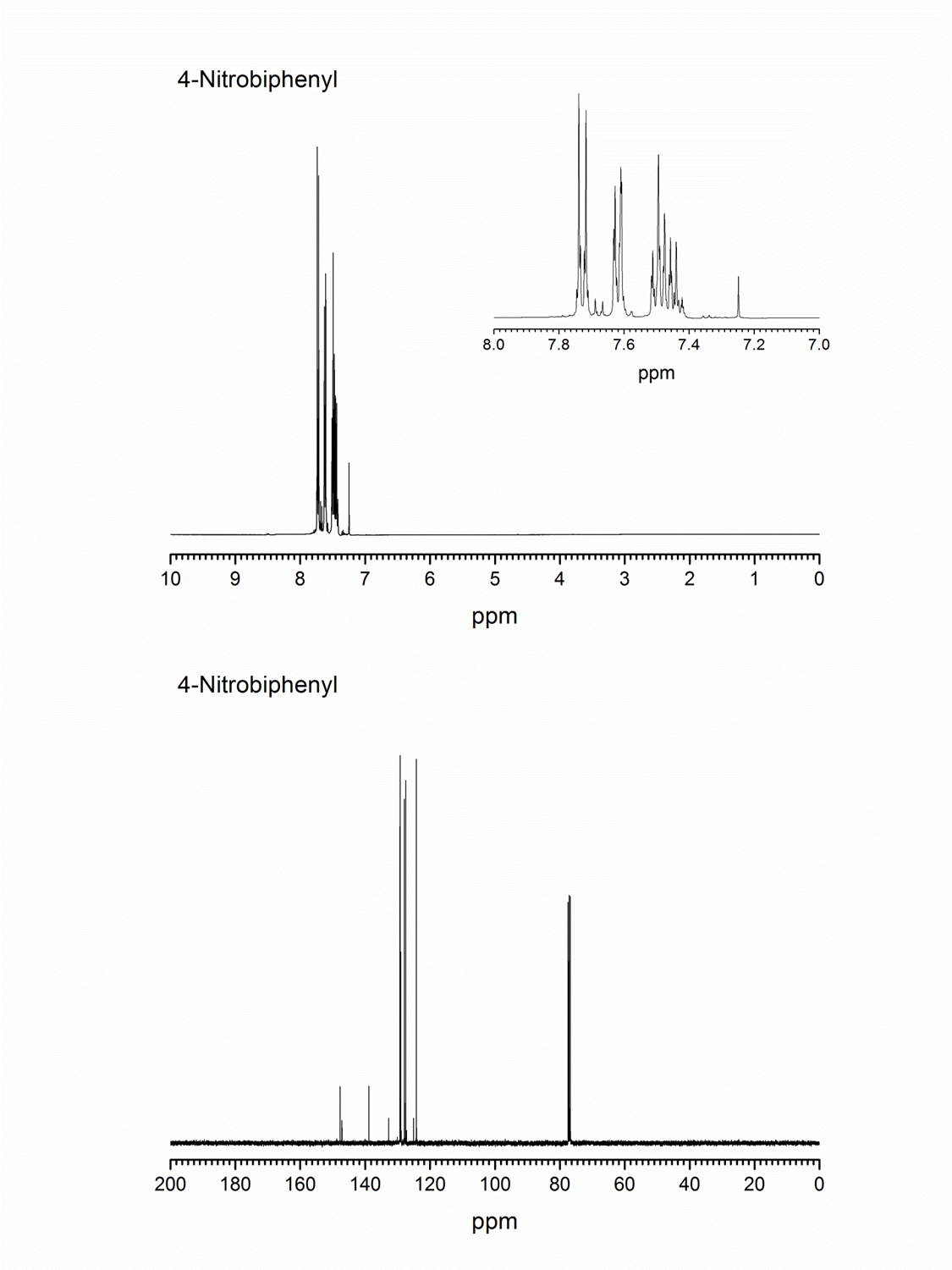

Supplement: Figure S8 — 4-Nitrobiphenyl 1H NMR and 13C NMR. (TIF) [file pone.0087192.s008.tif]

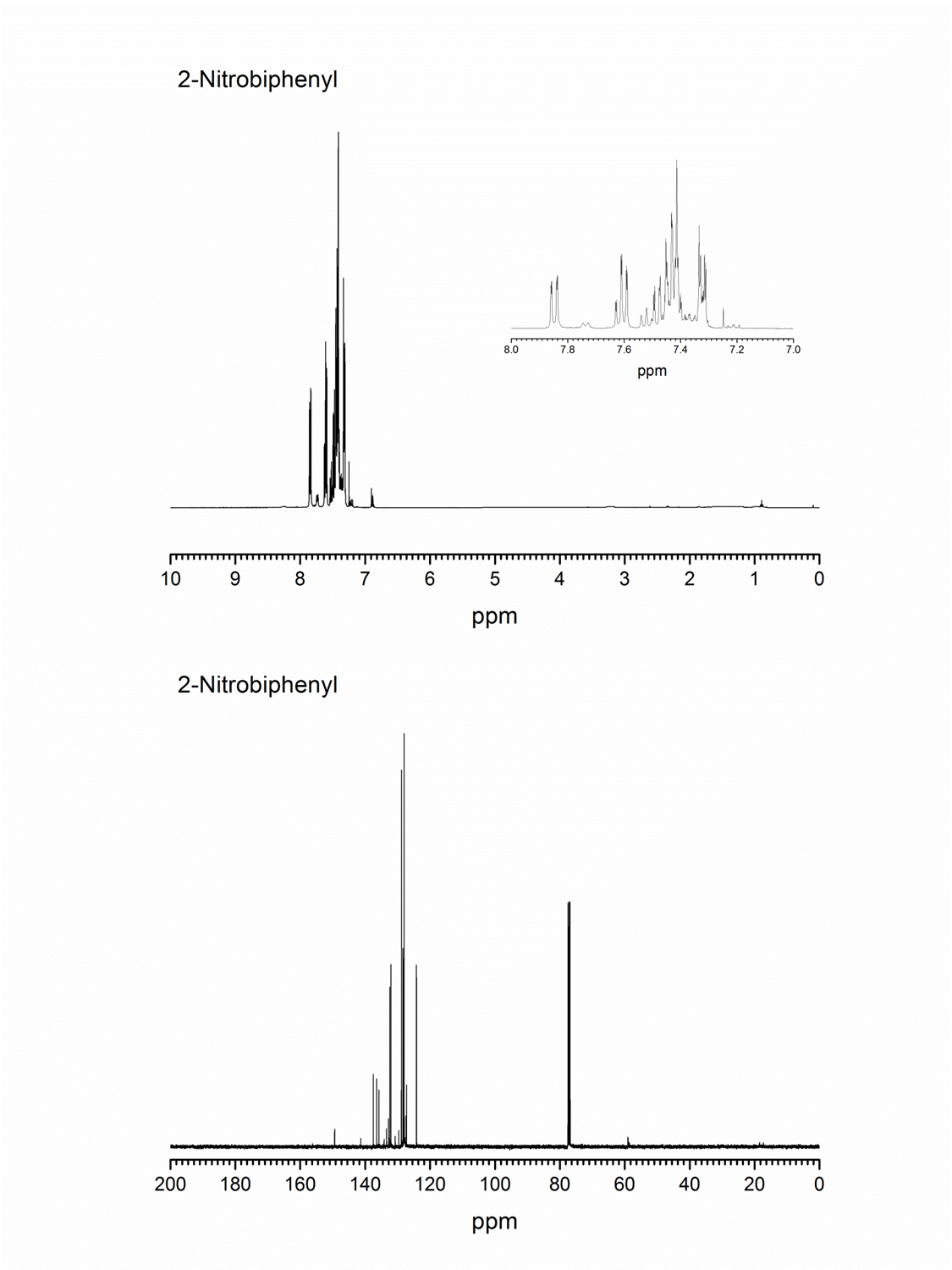

Supplement: Figure S9 — 2-Nitrobiphenyl 1H NMR and 13C NMR. (TIF) [file pone.0087192.s009.tif]

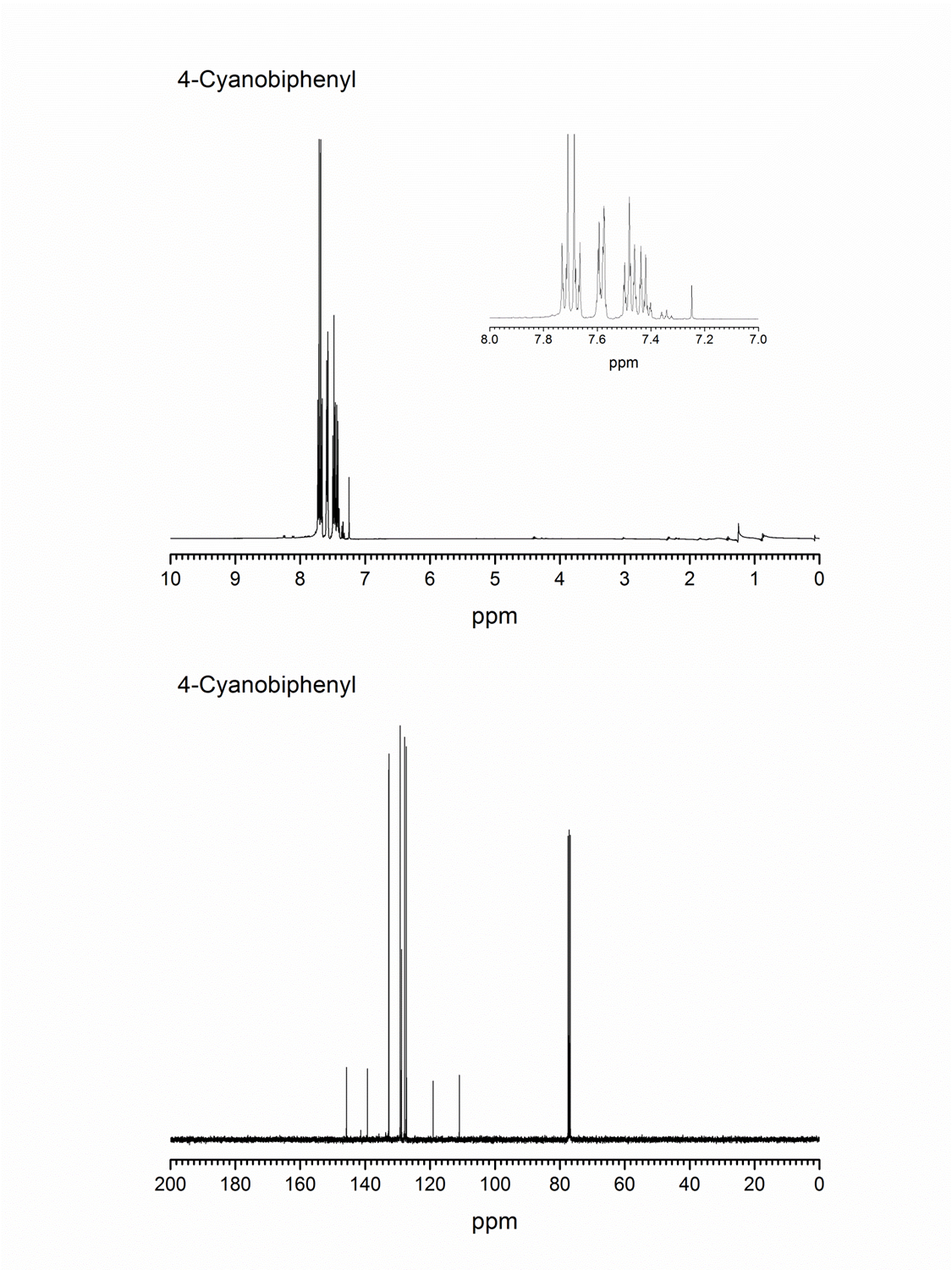

Supplement: Figure S10 — 4-Cyanobiphenyl 1H NMR and 13C NMR. (TIF) [file pone.0087192.s010.tif]

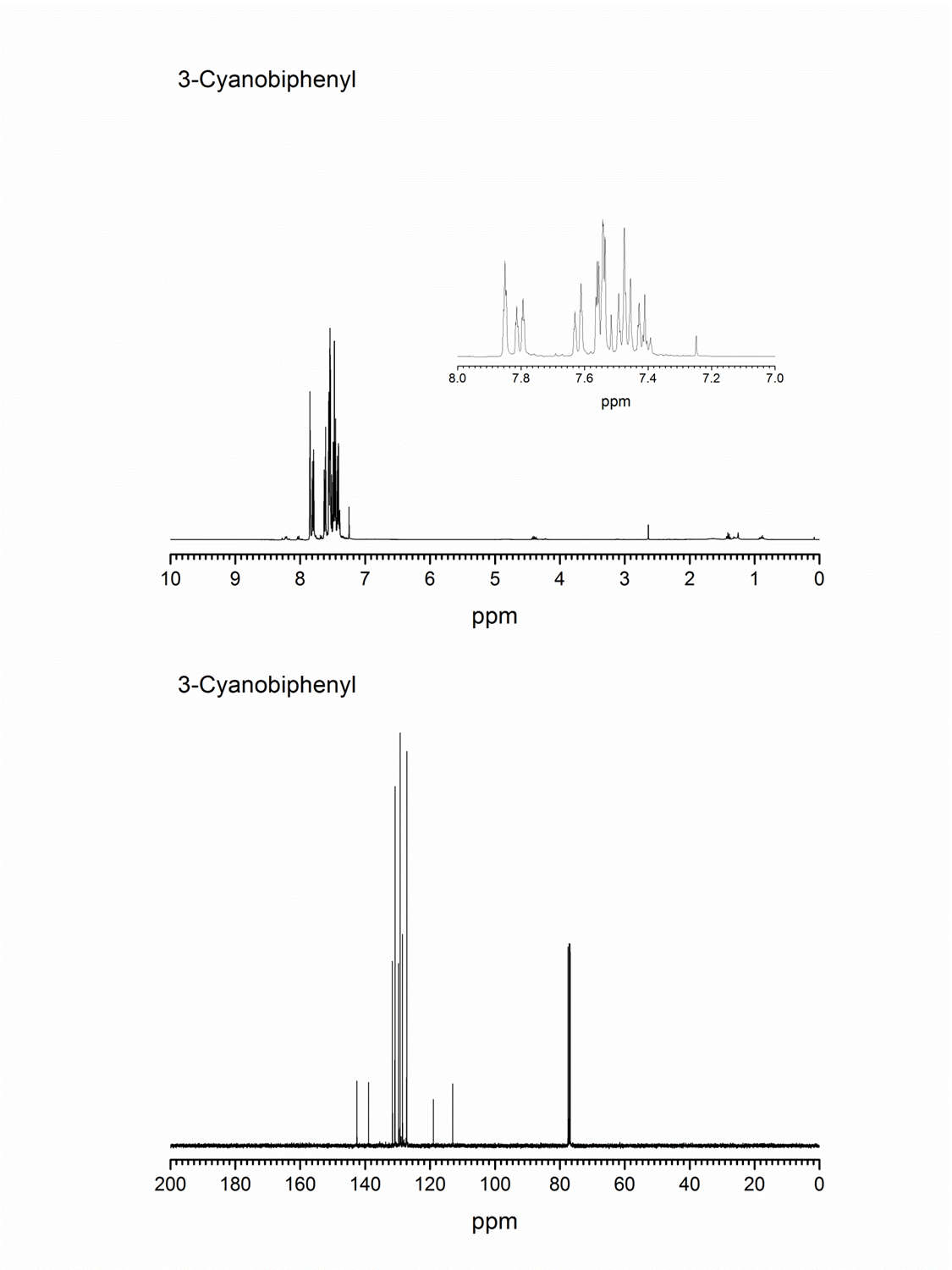

Supplement: Figure S11 — 3-Cyanobiphenyl 1H NMR and 13C NMR. (TIF) [file pone.0087192.s011.tif]
