# Supplementary material for: Supported Palladium Nanoparticles Synthesized by Living Plants as a Catalyst for Suzuki-Miyaura Reactions
Source: PLoS One. 2014 Jan 29;9(1):e87192. doi: 10.1371/journal.pone.0087192 (PMC3906157; doi:10.1371/journal.pone.0087192)
Supplement: Table S1 — Composition palladium present in carbonised palladium plant (Pd-P-300) from XPS spectra. (DOCX) [file pone.0087192.s012.docx]

**Table S1.** Composition palladium present in carbonised palladium plant (Pd-P-300) from XPS spectra.

|  | **Peak** | **BE [eV]** | **Chemical state** | **%** | |
| --- | --- | --- | --- | --- | --- |
|  |  |  |  | **Pd-P-300** | **Pd/C** |
| Palladium | I | 334.5 | Pd^(0)^ | 14 | - |
|  | II | 336.8 ± 0.2 | PdO | 42 | 58 |
|  | III | 340.0 ± 1.0 | Pd^(2+)^ | 44 | 42 |
