# Supplementary material for: Supported Palladium Nanoparticles Synthesized by Living Plants as a Catalyst for Suzuki-Miyaura Reactions
Source: PLoS One. 2014 Jan 29;9(1):e87192. doi: 10.1371/journal.pone.0087192 (PMC3906157; doi:10.1371/journal.pone.0087192)
Supplement: Table S2 — Screening conditions to find optimal conditions for Suzuki reactions. (DOCX) [file pone.0087192.s013.docx]

**Table S2.** Screening conditions to find optimal conditions for Suzuki reactions.

| **Entry** | **Base** | **Solvent** |
| --- | --- | --- |
| 1 | K_2_CO_3_ | Ethanol |
| 2 |  | Water |
| 3 |  | Ethanol:Water |
| 4 | Na_2_CO_3_ | Ethanol |
| 5 |  | Water |
| 6 |  | Ethanol:Water |
| 7 | Triethylamine | Ethanol |
| 8 |  | Water |
| 9 |  | Ethanol:Water |
