# Supplementary material for: Supported Palladium Nanoparticles Synthesized by Living Plants as a Catalyst for Suzuki-Miyaura Reactions
Source: PLoS One. 2014 Jan 29;9(1):e87192. doi: 10.1371/journal.pone.0087192 (PMC3906157; doi:10.1371/journal.pone.0087192)
Supplement: Table S3 — 1H NMR and 13C NMR data for isolated products. (DOCX) [file pone.0087192.s014.docx]

# Table S3. ^1^HNMR and ^13^CNMR data for the products.

| **Product** | **^1^H NMR and ^13^C NMR data** |
| --- | --- |
| Biphenyl | ^1^HNMR (400 MHz, CDCl_3_, ppm): 7.69-7.61 (m, 4H), 7.48-7.44 (m, 4H), 7.41-7.40 (m, 2H). ^13^CNMR (100 MHz, CDCl_3_, ppm): 140.0, 129.0, 128.3, 127.4, 127.3. |
| 4-Chlorobiphenyl | ^1^HNMR (400 MHz, CDCl_3_, ppm): 7.57-7.50 (m, 4H), 7.46-7.35 (m, 5H). ^13^CNMR (100 MHz, CDCl_3_, ppm): 140.1, 139.8, 132.8, 129.0, 128.5, 128.1, 127.7, 127.1. |
| 4-Acetylbiphenyl | ^1^HNMR (400 MHz, CDCl_3_, ppm): 8.03-8.01 (dd, 2H, *J* = 2, 6.6 Hz), 7.69-7.67 (dd, 2H, *J* = 2, 6.6 Hz), 7.63-7.61 (m, 2H), 7.48-7.44 (m, 2H), 7.41-7.40 (m, 1H), 2.63 (s, 3H). ^13^CNMR (100 MHz, CDCl_3_, ppm): 197.8, 145.9, 140.0, 135.9, 129.0, 128.3, 127.4, 127.3, 26.8. |
| 3-Nitrobiphenyl | ^1^HNMR (400 MHz, CDCl_3_, ppm): 8.46 (s, 1H), 8.21-8.19 (d, 1H, *J* = 8 Hz), 7.92-7.90 (d, 1H, *J* = 8 Hz), 7.65-7.59 (m, 3H), 7.52-7.26 (m, 3H). ^13^CNMR (100 MHz, CDCl_3_, ppm): 148.7, 142.8, 138.6, 133.0, 129.7, 129.1, 128.5, 127.1, 122.0, 121.9. |
| 2-Acetylbiphenyl | ^1^HNMR (400 MHz, CDCl_3_, ppm): 7.82-7.70 (m, 4H), 7.59-7.46 (m, 5H), 2.39 (s, 3H). ^13^CNMR (100 MHz, CDCl_3_, ppm): 204.7, 141.1, 140.9, 130.9, 130.4, 129.0, 128.9, 128.1, 128.0, 127.6, 30.6. |
| 4-Nitrobiphenyl | ^1^HNMR (400 MHz, CDCl_3_, ppm): 7.73-7.66 (m, 2H), 7.60-7.57 (m, 2H), 7.48-7.42 (m, 5H). ^13^CNMR (100 MHz, CDCl_3_, ppm): 145.8, 139.2, 132.7, 129.2, 128.8, 127.8, 127.3, 119.1, 111.0. |
| 2-Nitrobiphenyl | ^1^HNMR (400 MHz, CDCl_3_, ppm): 7.86-7.84 (dd, 1H, *J* = 1.3, 8 Hz), 7.63-7.59 (td, 1H, *J* = 1.3, 8 Hz), 7.54-7.40 (m, 5H), 7.33-7.31 (m, 2H). ^13^CNMR (100 MHz, CDCl_3_, ppm): 149.4, 137.5, 136.4, 132.4, 132.1, 128.8, 128.3, 128.1, 127.4, 124.2. |
| 4-Cyanobiphenyl | ^1^HNMR (400 MHz, CDCl_3_, ppm): 7.73-7.65 (m, 4H), 7.60-7.57 (m, 2H), 7.53-7.41 (m, 3H). ^13^CNMR (100 MHz, CDCl_3_, ppm): 145.8, 141.3, 139.3, 135.7, 134.0, 132.7, 129.8, 129.3, 128.9, 127.8, 127.3, 120.5, 115.4. |
| 3-Cyanobiphenyl | ^1^HNMR (400 MHz, CDCl_3_, ppm): 7.86-7.79 (m, 2H), 7.63-7.39 (m, 7H). ^13^CNMR (100 MHz, CDCl_3_, ppm): 142.5, 139.0, 131.6, 130.8, 129.7, 129.2, 128.5, 127.2, 119.0, 113.0. |
